# High myopia is protective against diabetic retinopathy in the participants of the National Health and Nutrition Examination Survey

**DOI:** 10.1186/s12886-023-03191-x

**Published:** 2023-11-17

**Authors:** Weijung Ten, Ying Yuan, Wei Zhang, Yue Wu, Bilian Ke

**Affiliations:** 1 Department of Ophthalmology, Shanghai General Hospital, Shanghai Jiao Tong University School of Medicine, No. 100 Haining Road, Shanghai, 200080 China; 2 Shanghai Key Laboratory of Fundus Disease, Shanghai, China; 3 Shanghai Engineering Center for Visual Science and Photomedicine, Shanghai, China; 4 Eye Institute and Department of Ophthalmology, Eye & ENT Hospital,, Fudan University, Shanghai, China; 5 Shanghai Public Health Clinical Center & Institutes of Biomedical Sciences, Fudan University, Shanghai, China

**Keywords:** High myopia, Diabetic retinopathy, NHANES

## Abstract

**Purpose:**

To investigate the association of subjects with refractive error and diabetic retinopathy (DR) in the United States comparing results between different race groups.

**Methods:**

All data were derived from National Health and Nutrition Examination Survey (NHANES) from 2005 to 2008. The data were divided into four groups (emmetropia, mild myopia, high myopia, hypertropia) according to the spherical equivalent (SE), and those who met the enrollment conditions were selected as the study subjects. Multivariable logistic regression analysis was used to evaluate the relationship between refractive error and diabetic retinopathy risk.

**Results:**

A total of 1317 participants were included in the study, including 331 participants with diabetic retinopathy, and 986 without diabetic retinopathy. After adjustment for potential confounders, subjects with high myopia were associated with a lower risk of diabetic retinopathy. The odds ratio (OR) was 0.44, 95% confidence interval (CI): (0.20–0.96), *P*-value = 0.040 in the multivariate regression analysis. Subgroup analyses showed that subjects with high myopia in the non-Hispanic Black group were associated with decreased odds of diabetic retinopathy. (OR was 0.20, and 95% CI: 0.04–0.95, *P*-value = 0.042).

**Conclusion:**

The results show that high myopia is associated with diabetic retinopathy in diabetic patients.

## Introduction

Diabetic retinopathy is one of the leading causes of blurred vision in middle-aged and elderly patients [1]. Irreversible central and peripheral vision loss can be triggered when effective treatment is not timely [2, 3]. Epidemiological studies have identified a variety of important systemic factors that influence the development of diabetic retinopathy, such as kidney damage, duration of diabetes mellitus, glycemic control, insulin use, hypertension, and cardiovascular disease [4–6]. Furthermore, several studies have mentioned that myopia is one of the factors that are protective against diabetic retinopathy. The correlation of these two eye diseases has been studied for over half a century [7–14]. Although the pathogeneses are different, the progressing development of high myopia and diabetic retinopathy both contribute to high risks of visual impairment. Through complete fundus examination (OCT, fundus photos), ophthalmologists can accurately determine the development of high myopia and diabetic retinopathy [15]. However, these studies focus on the analysis of the correlation between patients with diabetic retinopathy and those with low or moderate myopia, and rarely analyze patients with high myopia. In addition, there are far more Asian population studies than Western population studies. To more accurately explore the relationship between diabetic retinopathy and different refractive degrees in western different races, we analyzed the data from the NHANES dataset.

## Materials and methods

### Data source and study population

The National Health and Nutrition Examination Survey (NHANES) is a survey of the noninstitutionalized civilian US population conducted by the Centers for Disease Control since 1959. Representative participants were randomly selected through a multistage sampling design to assess their health and nutritional status. According to the 1975 Helsinki Declaration, all participants provided informed consent before enrollment.

**Fig. 1:**
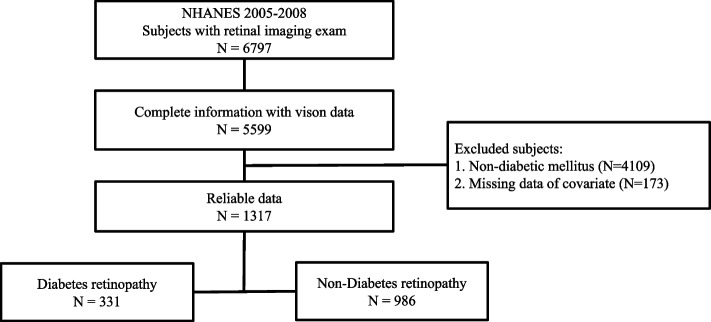
Flowchart of studied participants selection

Two study protocols of NHANES (2005–2008) were collected in our analysis. The flow diagram of the participation is presented in Fig. 1. A total of 6797 participants aged ≥ 40 years had retinal photographs and were obtained. We excluded those with incomplete retinopathy information and vision data (*n* = 1198), no diabetes mellitus (*n* = 4109), and absence of covariates (*n* = 173). 1317 subjects were ultimately included in this study.

### Definition of diabetic retinopathy

Participants sat in a room with no windows and lights off, and two images of the fundus exam were obtained using the Canon EOS 10D digital camera (Canon, Tokyo, Japan) and the Canon CR6-45N Ophthalmic Digital Imaging System. The pictures provided photographic documentation of the optic disc, macula, and substantial portions of the temporal arcades. Diabetic retinopathy was diagnosed by ophthalmologists at the University of Wisconsin. Researchers evaluated grading standards of diabetic retinopathy based on the Early Treatment for Diabetic Retinopathy Study (ETDRS). In the study, diabetic retinopathy was defined as the presence of retinal microaneurysms, blot hemorrhage, hard exudate, soft exudate, intraretinal microvascular abnormalities, venous beading, or fibrous proliferation. Also, clinically significant macular edema (CSME) on photographs in patients with diabetes was considered as diabetic retinopathy.

### Assessment of refractive error

Objective refraction of both eyes was assessed using the Nidek Autorefractor Model ARK-760, without cycloplegia or pupillary dilation. The data of measurements were converted into SE calculated as the spherical value plus half of the astigmatic value. Emmetropia was defined as the average SE value from -1.0 D to 1.0 D, mild myopia from -1.0 D to -5.0 D, high myopia as less than or equal to -5.0 D, and hypertropia as more than 1.0 D.

### Ascertainment of diabetes mellitus

The definition of Diabetic Mellitus in the NHANES study was described previously. According to the criteria, (1) Diabetic Mellitus was ascertained by the self-reported history of a physician diagnosis DIQ010 “Doctor told you to have diabetes” in the “Diabetes” questionnaire section in NHANES. The answer “yes” represented Diabetic Mellitus; (2) glycated hemoglobin A1c (HbA1c) ≥ 6.5%; (3) received antidiabetic treatments including insulin or oral hypoglycemic agents.

### Other variables

Demographic characteristics were collected from a computer-assisted personal interview system including age, gender, race/ethnicity (non-Hispanic white, non-Hispanic Black, Hispanic, other), and education attainment (high school or below, any college). Body mass index (BMI) was calculated as weight in kilograms divided by height in meters squared. The family poverty income ratio (PIR) compares family income to US census-defined poverty levels. The CVD-related information was assessed by a self-reporting questionnaire of coronary heart disease, angina, myocardial infarction, stroke, or congestive heart failure. Cigarette smoking status was attained by using the questions “Do you now smoke cigarettes?”, and “Smoked at least 100 cigarettes in life?”.

### Statistical analysis

We obtained and merged the data from the 2005–2008 NHANES as mentioned previously. For the description of the baseline characteristics, categorical variables and continuous variables were performed using the chi-squared test and t-test for proportions and means, respectively.

Three logistic regression models were used to evaluate the relationship between high myopia and diabetic retinopathy, and the adjusted ORs and 95% CI were calculated. Model I was unadjusted regression. Next, Model II was adjusted for sex, age, and race/ethnicity. Finally, Model III was further adjusted for education level, BMI, PIR, CVD, hypertension, and smoking based on Model II. All statistical analyses were conducted using SAS (version 9.4). All tests were two-sided with a significance level of *p* < 0.05.

## Result

A total of 1317 participants met the criteria after excluding missing data of gradable fundus photographs, incomplete vision data, and non-diabetics. Among them were 986 subjects with non-diabetic retinopathy, and 331 subjects with diabetic retinopathy. In addition, there were 610 subjects in the total study population with refractive errors (46.32%).

### Characteristics of the study population

The demographic characteristics of subjects in the study population with and without high myopia are described in Table 1. Compared to the subjects in the non-diabetic retinopathy group, those with diabetic retinopathy tend to have lower PIR levels (2.57 ± 1.59 vs. 2.31 ± 1.44 kg/m^2^, *P* = 0.010). There were more Female subjects in the diabetic retinopathy group than in the non-diabetic retinopathy group (56.50% vs. 48.68%, *P* = 0.014). Participants with diabetic retinopathy were likely to have CVD (26.89% vs. 17.34%, *P* =  < 0.001).

**Table 1:** Baseline characteristics in unmatched subjects of 2005–2008 NHANES

Variables	Non-DR	DR	*P*
	*n* = 986	*n* = 331	
Age (years)	61.90 ± 11.15	63.53 ± 10.72	0.020
PIR	2.57 ± 1.59	2.31 ± 1.44	0.010
BMI (kg/m^2^)	32.07 ± 7.08	31.45 ± 6.83	0.165
Sex			0.014
Male	506 (51.32%)	144 (43.50%)	
Female	480 (48.68%)	187 (56.50%)	
Race/ethnicity			< 0.001
Non-Hispanic White	472 (47.87%)	124 (37.46%)	
Non-Hispanic Black	236 (23.94%)	117 (35.35%)	
Hispanic	244 (24.75%)	83 (25.08%)	
Other Races	34 (3.45%)	7 (2.11%)	
Education			0.031
< College	599 (60.75%)	233 (67.37%)	
> College	287 (39.25%)	108 (32.63%)	
Smoke			0.075
No	433 (43.91%)	164 (49.55%)	
Yes	553 (56.09%)	167 (50.45%)	
CVD			< 0.001
No	815 (82.66%)	242 (73.11%)	
Yes	171 (17.34%)	89 (26.89%)	
Refractive errors			0.002
Emmetropia (-1.0D < SE < 1.0D)	407 (41.28%)	134 (40.48%)	
Mild myopia (-5.0D < SE < -1.0D)	383 (38.84%)	157 (47.43%)	
High myopia (SE < -5.0D)	62 (6.29%)	8 (2.42%)	
Hyperopia (1.0D < SE)	134 (13.59%)	32 (9.67%)	

*NHANES* National Heal sth and Nutrition Examination Survey, *SE* Spherical equivalent, *DR* Diabetic retinopathy, *BMI* Body mass index, *CVD* Cerebrovascular disease, *PIR* Poverty impact ratio

Continuous variables: mean ± standard deviation; categorical variables: frequency (%)

### Associations of refractive error with diabetic retinopathy

Among the participants in the present study, diabetic retinopathy compared with non-diabetic retinopathy was associated with a lower risk of high myopia, and the association was not significant between other refractive degrees and diabetic retinopathy. The results from the multivariate logistic regression were shown in Table 2. In unadjusted analysis (Model I), subjects with high myopia had an OR of 0.39 (95% CI: 0.18–0.84, *P*-value = 0.016) for diabetic retinopathy. After adjusting for age, sex, and race/ethnicity (Model II), the results were significantly unchanged. The OR (95% CI) of diabetic retinopathy was 0.43 (95% CI: 0.20–0.93, *P*-value = 0.032) in the high myopia patients. After additional adjustments including education, BMI, PIR, CVD and smoke (Model III), the association between high myopia and diabetic retinopathy remained significant. (OR: 0.44, 95%CI: 0.20–0.96, *P*-value = 0.040).

**Table 2:** Association of high myopia with diabetic retinopathy

	Model I		Model II		Model III	
	OR 95%CI	*P*	OR 95%CI	*P*	OR 95%CI	*P*
Emmetropia (-1.0D < SE < 1.0D)	Reference		Reference		Reference	
Mild myopia (-5.0D < SE < -1.0D)	1.25 (0.95–1.63)	0.111	1.24 (0.95–1.64)	0.119	1.28 (0.97–1.69)	0.084
High myopia (SE < -5.0D)	0.39 (0.18–0.84)	0.016	0.43 (0.20–0.93)	0.032	0.44 (0.20–0.96)	0.040
Hyperopia (1.0D < SE)	0.73 (0.47–1.12)	0.145	0.70 (0.45–1.10)	0.120	0.72 (0.46–1.13)	0.151

Model I: unadjusted

Model II: adjusted for age, sex, race/ethnicity

Model III: adjusted for age, sex, race/ethnicity, education, BMI, PIR, smoke and CVD

*BMI* Body mass index, *CVD* Cerebrovascular disease, *PIR* Poverty impact ratio, *DR* Diabetic retinopathy

### Multivariate Analysis for diabetic retinopathy and the Risk of high myopia in different races

In this multivariable analysis (Table 3), three models were adjusted for the same variables as Table 2. The correlation between diabetic retinopathy and high myopia persisted in the non-Hispanic Black group, while there was no association in other races. The OR of the non-Hispanic Black group was 0.20 (95%CI: 0.04–0.95, *P*-value = 0.042) after adjusting additional covariate including education, BMI, PIR, CVD, and smoke by Model III.

**Table 3:** Multivariate analysis for diabetic retinopathy and the Risk of high myopia in different races

	OR	95%CI	*P*-value
Non-Hispanic White			
Emmetropia (-1.0D < SE < 1.0D)	Reference		
Mild myopia (-5.0D < SE < -1.0D)	1.04	0.67–1.61	0.874
High myopia (SE < -5.0D)	0.45	0.13–1.56	0.208
Hyperopia (1.0D < SE)	0.97	0.48–1.98	0.936
Non-Hispanic Black			
Emmetropia (-1.0D < SE < 1.0D)	Reference		
Mild myopia (-5.0D < SE < -1.0D)	1.32	0.81–2.15	0.264
High myopia (SE < -5.0D)	0.20	0.04–0.95	0.042
Hyperopia (1.0D < SE)	0.41	0.17–1.01	0.053
Hispanic			
Emmetropia (-1.0D < SE < 1.0D)	Reference		
Mild myopia (-5.0D < SE < -1.0D)	1.52	0.86–2.70	0.154
High myopia (SE < -5.0D)	0.81	0.21–3.11	0.757
Hyperopia (1.0D < SE)	0.87	0.38–1.98	0.739
Other races			
Emmetropia (-1.0D < SE < 1.0D)	Reference		
Mild myopia (-5.0D < SE < -1.0D)	14.80	0.44–501.84-	0.134
High myopia (SE < -5.0D)	-	-	-
Hyperopia (1.0D < SE)	18.45	0.384–885.49	0.140

Adjusted for age, sex, education, BMI, PIR, smoke and CVD

*BMI* Body mass index, *CVD* Cerebrovascular disease, *PIR* Poverty impact ratio, *DR* Diabetic retinopathy

## Discussion

We used NHANES 2005–2008 data, which was collected from a public source with a complex multistage survey design for our cross-sectional study, and the results were consistent in different statistical models. After adjusting all the factors, we substantiated that the protective effect of high myopia was associated with a reduced likelihood of diabetic retinopathy (OR: 0.44, 95% CI: 0.18–0.96) In the stratified analyses, subjects with high myopia levels did have a significantly lower risk of diabetic retinopathy in the non-Hispanic Black group (OR: 0.20, 95%CI: 0.04–0.95). 

The prevalence of people with high myopia from this screening data was 5.69%, which is lower than the U.K. population (9.5%), but higher than the Chinese (3.93%) and elder Indian population (< 1%) [16–18]. Many epidemiological surveys had proposed that an increase in refractive is a protective influence against diabetic retinopathy. However, some studies showed conflicting results in severe myopia in meta-analysis studies [19–22].

In recent years, axial length has been considered to be one of the most important eye measurement standards for diagnosing myopia, and more than 26 mm can be regarded as high myopia [23, 24]. Therefore, the relationship between severe myopia and diabetic retinopathy was also assessed based on the axial length in some studies. Two Singaporean cohort studies had described that the OR value of subjects with diabetic retinopathy were 0.86 and 0.68 per 1 mm increase in axis length, respectively [7, 9]. As for the Chinese population in Beijing, Xu et al. explored the incidence of diabetic retinopathy and its related factors in 2602 participants during a ten-year study. The results showed a total of 109 new patients with diabetic retinopathy during the ten years. These patients had a shorter eye axis length than the non-diseased person (OR: 0.48; 95% CI: 0.33–0.71) [25]. The prevalence of diabetic retinopathy was not related to refractive measurements, but the long axial length was associated with lower diabetic retinopathy [12]. This conclusion was similar to another study of Chinese populations [26]. In the cross-section study, Wang et al. measured axial lengths as an eye parameter for the prevalence and severity of diabetic retinopathy. A higher prevalence of diabetic retinopathy is associated with shorter axial lengths (OR: 0.81, 95% CI: 0.70–0.95) [13]. Unfortunately, although longer axial length is closely related to diabetic retinopathy, the structural component of participants was not measured in the NHANES datasets. Thus, we could not discuss the influence of axial length in our analysis even if it's an important diagnostic indicator.

According to the above-mentioned, Myopia is widely considered to be one of the protective factors for DR, but most studies have explored the situation in Asia. Few studies have explored the relationship between high myopia and DR in different Western populations based on large sample sizes. We found that high myopia has a significant negative relationship with DR in black people. There was a negative correlation between high myopia interval and diabetic retinopathy in univariate analysis (OR: 0.39, 95% CI: 0.18–0.84). After adjustment for age, gender, and race (Model II), high myopia might reduce the risk of diabetic retinopathy compared with those in the emmetropia group (OR: 0.43, 95% CI: 0.20–0.93). After further adjustment for education level, BMI, PIR, CVD, and smoking status (Model III), high myopia was still associated with a reduced diabetic retinopathy risk (OR: 0.44, 95% CI: 0.20–0.96).

To the best of our knowledge, there are no large-scale population-based studies that have focused on the relationship between high myopia and diabetic retinopathy in Western countries. Therefore, we investigated NHANES, a database with a complex sampling design in the United States population, and found high myopia is regarded as a factor influencing diabetic retinopathy in this public database. Moreover, the correlation between different races and diabetic retinopathy may be an influence factor. Nwanyanwu et al. described the relationship between race and the prevalence of diabetic retinopathy [27]. In the present study, High myopia is a protective factor for diabetic retinopathy in black populations. 

Some limitations of this study should be acknowledged. First of all, the analysis was of cross-sectional design, thus our study cannot determine a causal relationship between two ocular diseases. Second, given the absence of data on the axial length of the eye in the NHANES dataset, we could not assess this main factor affecting refractive error. However, according to previous studies, some population-based studies also classify the degree of myopia solely based on SE value. If axial length assessment could be provided in later NHANES studies, combined with the SE classification method, it would provide more accurate measurements for myopia severity. Last, we had not analyzed the association between high myopia and proliferative diabetic retinopathy, which represents the last stage of this extremely complex retinal disease. This is because very few participants simultaneously suffered from high myopia and proliferative diabetic retinopathy in our analysis.

## Conclusion

Based on the analysis for adjusting potential confounding variables, we concluded that participants with high myopia have a lower risk of diabetic retinopathy in the U.S. population, and such a conclusion was also statistically significant in the non-Hispanic Black group. Future longitudinal studies are needed to promote an understanding of the relationship between high myopia and the progression of diabetic retinopathy.

## Data Availability

The datasets generated and analyzed during the current study are available in the NHANES database. https://wwwn.cdc.gov/Nchs/Nhanes/.
